# Extensive Recombination Suppression and Epistatic Selection Causes Chromosome-Wide Differentiation of a Selfish Sex Chromosome in *Drosophila pseudoobscura*

**DOI:** 10.1534/genetics.120.303460

**Published:** 2020-07-30

**Authors:** Zachary L. Fuller, Spencer A. Koury, Christopher J. Leonard, Randee E. Young, Kobe Ikegami, Jonathan Westlake, Stephen Richards, Stephen W. Schaeffer, Nitin Phadnis

**Affiliations:** * Department of Biology, The Pennsylvania State University, University Park, Pennsylvania 16802; † Department of Biological Sciences, Columbia University, New York, New York 10027; ‡ School of Biological Sciences, University of Utah, Salt Lake City, Utah 84112; § Department of Genetics, University of Wisconsin, Madison, Wisconsin 53706; ** Human Genome Sequencing Center, Baylor College of Medicine, 1 Baylor Plaza, Houston, Texas 77030

**Keywords:** meiotic drive, chromosomal inversions, recombination, selfish genetic elements

## Abstract

*Sex-Ratio* (*SR*) chromosomes are selfish *X*-chromosomes that distort Mendelian segregation and are commonly associated with inversions. These chromosomal rearrangements suppress recombination with Standard (*ST*) *X*-chromosomes and are hypothesized to maintain multiple alleles important for distortion in a single large haplotype. Here, we conduct a multifaceted study of the multiply inverted *Drosophila pseudoobscura SR* chromosome to understand the evolutionary history, genetic architecture, and present-day dynamics that shape this enigmatic selfish chromosome. The *D. pseudoobscura SR* chromosome has three nonoverlapping inversions of the right arm of the metacentric *X*-chromosome: basal, medial, and terminal. We find that 23 of 29 Mb of the *D. pseudoobscura* *X*-chromosome right arm is highly differentiated between the Standard and *SR* arrangements, including a 6.6 Mb collinear region between the medial and terminal inversions. Although crossing-over is heavily suppressed on this chromosome arm, we discover it is not completely eliminated, with measured rates indicating recombination suppression alone cannot explain patterns of differentiation or the near-perfect association of the three *SR* chromosome inversions in nature. We then demonstrate the ancient basal and medial inversions of the *SR* chromosome contain genes sufficient to cause weak distortion. In contrast, the younger terminal inversion cannot distort by itself, but contains at least one modifier gene necessary for full manifestation of strong sex chromosome distortion. By parameterizing population genetic models for chromosome-wide linkage disequilibrium with our experimental results, we infer that strong selection acts to maintain the near-perfect association of *SR* chromosome inversions in present-day populations. Based on comparative genomic analyses, direct recombination experiments, segregation distortion assays, and population genetic modeling, we conclude the combined action of suppressed recombination and strong, ongoing, epistatic selection shape the *D. pseudoobscura SR* arrangement into a highly differentiated chromosome.

## Abstract (precis)

The *Drosophila pseudoobscura Sex-Ratio* (SR) chromosome was one of the first-discovered segregation distorter chromosomes. Despite being a historically significant and well-studied segregation distortion system, the mechanisms allowing for the long-term....

*SEX-Ratio* (*SR*) chromosomes are selfish *X*-chromosomes that distort Mendelian segregation in their own favor by decreasing *Y*-bearing sperm (Policansky and Ellison 1970; Fredga *et al.* 1976; Hauschteck-Jungen and Maurer 1976; Gileva 1987; Montchamp-Moreau and Joly 1997; Wilkinson and Sanchez 2001). As a result, males that carry an *SR* chromosome produce an excess of female progeny. Such driving *X*-chromosomes have been detected in multiple species of both Drosophilidae and Diopsidae, and are commonly associated with chromosomal rearrangements (Gershenson 1928; Sturtevant and Dobzhansky 1936; Stalker 1961; Jungen 1967; de Carvalho *et al.* 1989; James and Jaenike 1990; Jaenike 1996, 2001; Presgraves *et al.* 1997; Yang *et al.* 2004; Tao *et al.* 2007; Unckless *et al.* 2015; Keais *et al.* 2017). Unchecked, these selfish genetic elements are expected to fix in populations, leading to eventual population extinction (Hamilton 1967; Wallace 1968; Lyttle 1977), yet *SR* chromosomes are often observed at stable frequencies and in some cases form long-term geographic clines (Sturtevant and Dobzhansky 1936; Dobzhansky 1944, p. 78; James and Jaenike 1990; Beckenbach 1996; Atlan *et al.* 1997; Dyer 2012; Verspoor *et al.* 2018). Furthermore, some *SR* chromosomes paradoxically have ancient origins but no suppressors, motivating interest in identifying the forces acting to maintain *SR* chromosomes across evolutionary timescales (Price *et al.* 2019). A number of mechanisms have been proposed to explain why *SR* chromosomes are not fixed or lost as populations go extinct, including the evolution of autosomal or *Y*-linked suppressors (de Carvalho and Klaczko 1994; Merçot *et al.* 1995; Carvalho *et al.* 1997; Atlan *et al.* 2003; Tao *et al.* 2007; Unckless *et al.* 2015), fitness differences among *X*-chromosome genotypes (Edwards 1961; Curtsinger and Feldman 1980; Wu 1983b; Holman *et al.* 2015; Larner *et al.* 2019), or a combination of differential sperm production, female remating rates, and sperm competition (Policansky and Ellison 1970; Policansky 1974; Price *et al.* 2008a,b, 2014). The molecular genetic mechanisms that underlie these distortion systems are largely unknown and may be quite diverse, therefore, both comparative genomic and classical genetic studies are a necessary first step in evaluating the forces that act on the origin, evolution and maintenance of *SR* chromosomes (Jaenike 1996; Dyer *et al.* 2007; Reinhardt *et al.* 2014; Fuller *et al.* 2018).

The *Drosophila pseudoobscura Sex-Ratio* (*SR*) chromosome represents one of the longest studied *Sex-Ratio* chromosomes (Sturtevant and Dobzhansky 1936; Dobzhansky 1944; Wu and Beckenbach 1983; Babcock and Anderson 1996). With respect to the wild type *Standard* (*ST*) *X*-chromosome, the *D. pseudoobscura SR* chromosome carries three nonoverlapping inversions on the right arm of the *X*-chromosome upon which all necessary and sufficient genes for the strong segregation distortion phenotype are located (Dobzhansky 1944, p. 78). *D. pseudoobscura* spermatocytes with *SR* chromosomes show normal segregation of the *X*- and *Y*-chromosomes in meiosis I; however, the *Y*-chromosome is found to be highly condensed at the metaphase plate of meiosis II and fails to segregate properly, such that these gametes fail to develop (Novitski *et al.* 1965). As a result, this *SR* chromosome distorts segregation ratios nearly completely, producing >99% *X*-bearing sperm (Policansky and Dempsey 1978). Moreover, it is found at frequencies approaching 30% in southwestern United States localities (Sturtevant and Dobzhansky 1936; Dobzhansky 1944, 1958). Despite the strong distortion and high frequency of *D. pseudoobscura SR*, no resistant *Y*-chromosomes or suppressor alleles have been identified even after extensive searches (Policansky and Dempsey 1978; Beckenbach *et al.* 1982). While *D. pseudoobscura SR* chromosomes are stereotypical of *SR* chromosomes more generally in their association with inversions (Stalker 1961; Jungen 1967; Voelker 1972; de Carvalho *et al.* 1989; Montchamp-Moreau and Cazemajor 2002; Dyer *et al.* 2007; Pieper and Dyer 2016) and their meiotic cytology (Poulson and Sakaguchi 1961; Fredga *et al.* 1976; Hauschteck-Jungen and Maurer 1976; Sweeny and Barr 1978; Gileva 1987; de Carvalho and Klaczko 1992; Jaenike 1996; Cazemajor *et al.* 1997, 2000; Wilkinson and Sanchez 2001); the absence of suppressors for an ancient, high frequency, strong distorter is unique and presents an evolutionary paradox (Stalker 1961; Voelker 1972; de Carvalho and Klaczko 1993; Helleu *et al.* 2015; Price *et al.* 2019).

Segregation distortion systems are often, but not always (*e.g.*, *Drosophila testacea*; James and Jaenike 1990), associated with low recombination regions of the genome, either within centromeric regions (Pimpinelli and Dimitri 1989; Cabot *et al.* 1993), inversions (Sturtevant and Dobzhansky 1936; Hammer *et al.* 1991; Dyer *et al.* 2007), or both (Larracuente and Presgraves 2012). Segregation distorters are thought to be closely associated with such regions because they can maintain groups of tightly linked alleles as a result of suppressed recombination with alternative arrangements, and maintain associations among multiple interacting loci required for distortion (Charlesworth and Hartl 1978; Lyon 2003; Kirkpatrick and Barton 2006; Dyer *et al.* 2007). For example, in the case of the autosomal segregation distortion (*SD*) system of *D. melanogaster*, distortion is the product of at least two interacting alleles: a driving locus (*Sd*) that causes distortion and a responder (*Rsp*) locus on which the driver can act (Hartl 1974; Wu *et al.* 1988; Larracuente and Presgraves 2012). Repeat number polymorphism at the *Rsp* locus is maintained in spite of distortion, where the high copy number *Rsp* alleles most sensitive to distortion are selectively advantageous compared to the low copy number insensitive *Rsp* alleles (Wu *et al.* 1989). In this segregation distortion system, population genetic analysis demonstrates that chromosomal inversions are favored because they prevent recombination between *Sd* and *Rsp*, thereby avoiding the formation of ‘suicide chromosomes’ (*i.e.*, when the distorter and sensitive responder alleles are found on the same chromosome, leading to self-destruction; (Hartl 1974; Charlesworth and Hartl 1978)). Thus, in models for autosomal segregation distortion, suppressed recombination acts to keep drivers and their targets linked in repulsion phase.

In contrast to autosomes, *X*- and *Y*-chromosomes do not recombine with each other in *Drosophila*, or generally in any brachycerous Dipterans (Gethmann 1988). Therefore, the prevention of suicide chromosomes is insufficient to explain the association of sex chromosome segregation distorters with chromosomal inversions. Because sex chromosome distortion fundamentally alters sex ratios, there is an intrinsic genetic conflict between *X*-linked, *Y*-linked, and autosomal loci that is hypothesized to fuel an ongoing evolutionary arms race between *X*-linked distorters, *Y*-linked resistance, and autosomal suppressors (Hamilton 1967; Thomson and Feldman 1975; Carvalho and Vaz 1999; Hurst and Werren 2001; Werren 2011). In this scenario, *SR* chromosome inversions act to establish tight linkage between epistatic alleles on the *X*-chromosome, permitting sex chromosome segregation distorters to persist by allowing the accumulation of alleles in coupling phase that either enhance distortion or help evade suppressors-of-distortion (Brittnacher and Ganetzky 1984; Jaenike 1996, 2001; Larracuente and Presgraves 2012). According to this idea, distorter systems that become associated with inversions enjoy an advantage by generating stronger drive mechanisms or by evading autosomal or *Y*-linked suppressors. Therefore, under this genetic conflict model, distorting chromosomes that are found with inversions are thought to evolve as large co-adapted gene complexes that accumulate and maintain epistatically interacting alleles to produce the distortion phenotype (Wu and Beckenbach 1983; Dyer *et al.* 2007).

Here, we perform a comparative genomic as well as experimental analyses of *SR* and *ST* strains of *D. pseudoobscura*. We first identified and confirmed breakpoints for two of three of the *SR* chromosomal inversions at a base-pair resolution. The breakpoint sequences display no obvious gene disruptions, chimeras, or transposable elements, indicating that the direct physical position effects of the inversions are unlikely to underlie the *SR* phenotype. Second, SNP divergence of breakpoint-flanking regions suggest that the *SR* chromosome is 813 ± 29 thousand years old, which is likely before or around the time of the split between *D. pseudoobscura* and *D. persimilis* (∼500 KYA; (Wang and Hey 1996; Tamura *et al.* 2004; Hey and Nielsen 2004)). This result is consistent with previous estimates based on the analysis of a single gene, *Esterase-5* (Babcock and Anderson 1996). We further estimate the relative ages of each of the three inversions, which suggest that the basal and medial inversions arose early around 800,000 years ago, whereas the terminal inversion arose ∼100,000 years later. Third, we find that 23 of 29 Mb of the XR chromosome arm is highly differentiated between the *ST* and *SR* arrangements, including the 6.6 Mb collinear region between the medial and terminal inversions. The pattern of polymorphism across these regions reject a simple neutral coalescent model of divergence, and requires more complex population dynamics involving selective forces to maintain genetic differences. This high level of differentiation includes a large number of fixed amino acid changes and differentially expressed genes across large regions of the *X*-chromosome, demonstrating that the *SR* arrangement provides a massive mutational target for the accumulation of fixed differences.

Transitioning to direct experiments, fourth, we show that recombination is largely suppressed – but not eliminated – in the 6.6 Mb collinear region between the medial and terminal inversions. Both population genetic and direct experimental evidence in this system show that recombination suppression extends into several megabases of the collinear sequence outside the inversion breakpoints. Fifth, we model the decay of linkage disequilibrium (LD) between *SR* chromosome inversions to demonstrate that strong past and ongoing epistatic selection is required to maintain high differentiation and the near perfect association of all three inversions in natural populations in the face of recombination. Finally, we isolated recombinant *SR* chromosomes and performed segregation assays. Contrary to previous anecdotal evidence (Dobzhansky 1944, p. 79; Wallace 1948; Beckenbach 1996), the basal and medial inversions in isolation are capable of driving, but we discovered that this drive is substantially reduced, whereas the terminal inversion alone is not capable of driving. Therefore, the strong unsuppressed distortion phenotype is expressed only when the weakly distorting locus in the older proximal half of the *SR* chromosome is paired with a modifier locus in the younger distal half of the *SR* chromosome. Combining our first result of the inversion ages with our last result of the segregation of recombinant *SR* chromosomes, suggests a historical scenario where *D. pseudoobscura SR* chromosomes evolve stronger drive by accumulating modifiers and additional inversions to bind these epistatic alleles in tight coupling phase LD. All together our analyses indicate the accumulation of genetic differences and maintenance of high differentiation across all three inversions of the *SR* chromosome requires an evolutionary model that incorporates the combined action of suppressed recombination and strong, ongoing selection enhancing segregation distortion.

## Materials and Methods

### Collection, isolation, and maintenance of *SR* chromosome strains

We collected wild *D. pseudoobscura* flies from Zion National Park, UT in September 2013 using bait consisting of an assortment of rotten fruits and screened them for the presence of *SR* chromosomes. Individual wild males collected were crossed to females from *ST D. pseudoobscura* stock with multiple markers on the *X*-chromosome: *cut*^*1*^ (*ct*^*1*^, 1–22.5), *scalloped^1^* (*sd*^*1*^, 1– 43), *yellow* (*y^1^*, 1–74.5) and *sepia*^*1*^ (*se*^*1*^, 1–145.1) (Orr and Irving 2001). Males carrying a *SR* chromosome are readily identified as those that produce nearly all female progeny. To screen for *SR* chromosomes in females, we allowed individual wild-caught females to produce progeny in the laboratory. The resulting sons were individually crossed to *ct*, *sd*, *y*, *se* females. Males carrying *SR* chromosomes were similarly identified as those that produced nearly all female progeny. We bred and tested a total of 113 *D. pseudoobscura* individuals, consisting of 66 males and 47 females. Of the 66 males collected and screened, 5 had an *SR* chromosome. Of the 47 females collected, 10 carried an *SR* chromosome. Of 160 *D. pseudoobscura X*-chromosomes tested (66 from males, 94 from females), 145 were *ST* chromosomes and 15 were *SR* chromosomes; *i.e.*, *SR* chromosomes we found at a frequency of ∼9.4% in this population. Once *SR* males were identified, we generated homozygous *SR* females using the *sepia* marker, which is known to cover the basal inversion on the *SR* chromosome (Babcock and Anderson 1996). All stocks were raised on standard cornmeal media at 18° C.

### DNA extractions and sequencing

To generate whole genome shotgun sequencing libraries for *D. pseudoobscura* strains, we first pooled one male each from 8 *SR* strains and 8 *ST* strains from our Zion National Park collections. We extracted DNA from these flies using the 5 Prime Archive Pure DNA extraction kit according to the manufacturer’s protocol (ThermoFisher, Waltham, MA). All libraries were generated with the Illumina TruSeq Nano kit (Epicentre, Illumina Inc., CA) using the manufacturers protocol, and sequenced as 500 bp paired end reads on an Illumina HiSeq 2000 instrument.

### Sequence alignment and SNP identification

Low-quality bases were removed from the ends of the raw paired-end reads contained in FASTQ files using *seqtk* (https://github.com/lh3/seqtk) with an error threshold of 0.05. Illumina adapter sequences and polyA tails were trimmed from the reads using Trimmomatic version 0.30 (Bolger *et al.* 2014). The read quality was then inspected manually using FastQC. Following initial preprocessing and quality control, the reads from each pool were aligned to the *D. pseudoobscura* reference genome (v 3.2) (Thurmond *et al.* 2019) using *bwa* version 0.7.8 with default parameters (Li and Durbin 2010). Of the total reads, 95.82% and 94.87% mapped successfully for the *ST* and *SR* pools, respectively (Supplemental Material, Table S1). Genome wide, the average fold coverage was ∼74x and ∼75x for the *D. pseudoobscura ST* and *SR* pools, respectively. For *X*-chromosome scaffolds, the average fold coverage was ∼45x and ∼46x (Table S2).

After the binary alignments were sorted and indexed with SAMtools (v. 0.1.19) (Li *et al.* 2009), Picard (v2.18.20; https://github.com/broadinstitute/picard) was used to mark duplicates and add read groups for each pooled sample. We called variants using GATK HaplotypeCaller (v3.8; McKenna *et al.* (2010), with the ploidy set separately for *X*-chromosome scaffolds (1*N*) and autosomes (2*N*). The GVCF files generated from HaplotypeCaller contain records for every genomic position, allowing us to set coverage thresholds for both variant and invariant sites. We considered any position with coverage less than the pool size and >3x SD from the mean coverage for that scaffold (Table S2) as missing, and therefore uncallable. Variants were filtered according to GATK’s hard filtering recommendations and we further masked calls ±5 bp surrounding indels. We then removed all multi-nucleotide polymorphisms to restrict our analyses to only biallelic SNPs. In total, 603,412 biallelic SNPs were called on chromosome *XR*. Sequences are deposited on the NCBI Short Read Archive (SRA) with accession numbers SRR6331544 and SRR6331545.

### Identifying and confirming the inversion breakpoints

We located the inversion breakpoints for the first two inversions of the *D. pseudoobscura* *SR* chromosome by viewing the mapped paired end reads of the *ST* and *SR* pooled genome sequences in the Integrated Genomics Viewer application using two methods. (1) We interpret the mapped paired end reads by pair orientation, such that parallel mapped paired-end reads where the read pair is mapped farther than expected and in the same orientation in the *SR* sequence but not the *ST* sequence is a clear indication that an inversion breakpoint is present. (2) Our sequencing library was prepared using 500 bp paired-end reads. When mapped paired end reads are located ∼500 bp from each other in the *ST* strains, but map over 1 Mb in *SR* strains, this is a clear indication that an inversion breakpoint is at that location (Corbett-Detig *et al.* 2012).

Inversion breakpoints were confirmed molecularly through a polymerase chain reaction (PCR) inversion assay. For proximal breakpoints, the forward primer is common to *ST* and *SR* with the reverse primer unique to *ST* or *SR*. For distal breakpoints, the forward primer is unique to *ST* or *SR* and the reverse primer is common to both *ST* and *SR*. For primers unique to *SR*, they were designed ∼500 bp from the opposite inversion breakpoint (if designed for the proximal breakpoint, primers were designed 500 bp before the distal breakpoint).

### Estimates of differentiation and divergence

To estimate population differentiation (*F_ST_*), we used the R package *poolfstat* which implements the methods-of-moments estimator developed by Hivert *et al.* (2018) and includes a correction for pooled sequencing. This estimator has been demonstrated to be unbiased and outperforms previous methods designed to estimate *F_ST_* in pooled sequencing data. To calculate confidence intervals (CIs), we modified the *computeFst* function to allow for sampling with replacement over each window. Divergence time estimates were taken with the Cavalli-Sforza (1969) transformation of *F_ST_* as

$$T=-log\;\left(1-F_{ST}\right)$$

and then multiplied by a scaling factor in each window so that the divergence time between *ST* and *D. miranda* was 2 MYA (Fuller *et al.* 2018).

Absolute sequence divergence was estimated with *d_XY_*, a measure of the number of pairwise nucleotide substitutions (Nei and Li 1979; Nei 1987). Following Love *et al.* (2016), we generated consensus reference sequences for each pooled sample by incorporating alleles probabilistically according to their frequency. The 95% CIs were similarly obtained by performing 10,000 bootstrap replicates across each region of interest. Custom Python code used to estimate *d_XY_* and generate the consensus sequences, as well as all R scripts used for plotting and statistical analyses are available at https://github.com/zfuller5280/Dpse_SR_analyses.

### Estimates of polymorphism and coalescent simulations

We estimated pairwise nucleotide diversity (*π*) and Tajima’s *D* in the pooled sequence data using PoPoolation (Kofler *et al.* 2011a). For each measure, we set the minimum allele count to 2, and the pool size equal to the number of chromosomes sequenced in each sample. To estimate polymorphism in nonoverlapping 10 kb intergenic regions, we used *bedtools* (v2.27.1; Quinlan and Hall (2010) to generate the genomic coordinates of regions located between annotated gene features extracted from the *gff* file in the genome assembly. Uncallable variant and invariant sites were removed from the calculation of polymorphism measures. By default, PoPoolation ignores windows where >40% of sites are uncallable. We further estimated *π* per-synonymous and per-nonsynonymous site (*i.e.*, *π_S_* and *π_N_*) using the software package SNPGenie (Nelson *et al.* 2015). SNPGenie was run separately for genes transcribed on the positive and negative strand. We restricted this analysis to protein-coding transcripts that were completely nonoverlapping, contained proper start and stop codons, and had a total length that was a multiple of 3.

To assess deviations of polymorphism levels from expectations under a simple neutral model of divergence and drift, we used coalescent simulations implemented with *msprime* (v0.7.6; Kelleher *et al.* (2016). Here, we simulated three lineages corresponding to *D. miranda*, *ST* and *SR*, and sampled 1, 8, and 8 chromosomes, respectively, from each at the present. We represented an inverted region by conservatively assuming no recombination between lineages (Pieper and Dyer 2016; Lotterhos 2019). Moreover, we assumed the frequency of the inversion reached equilibrium instantaneously after arising by sampling a single chromosome from the parental population (White *et al.* 2007). We specified the total *N_e_* of *D. pseudoobscura* as 1.8 × 10^6^ (Haddrill *et al.* 2010) and the *N_e_* of *D. miranda* as 3.6 × 10^5^, corresponding to a fivefold reduction that has been estimated previously (Yi *et al.* 2003). We considered three different *N_e_* values for *SR* based on current observed frequencies in natural populations across its range. First, we used a value of *N_e_* equal to 30% of the population based on the maximum observed frequency. Second, we used a value of *N_e_* equal to 13.5% of the population, corresponding to the mean observed frequency (see Table 1). Lastly, we used a value of *N_e_* equal to 1% of the total population to represent the minimum observed frequency in nature. In all cases, the *N_e_* of *ST* was adjusted so the total *D. pseudoobscura N_e_* remained 1.8 × 10^6^. All simulations assumed that *SR* arose from *ST* with a single event 2 million generations ago, and that *ST* diverged from *D. miranda* in a single event twice as old (*i.e.*, 4 million generations). Additionally, we considered simulations where the *SR–ST* divergence occurred 4 million generations ago and the *ST–D. miranda* divergence occurred 8 million generations ago, and none of the qualitative conclusions changed. All values of *N_e_* were multiplied by 3/4 to account for hemizygosity on the *X*-chromosome. We used a mutation rate of 3.5 × 10^−9^ per-base-pair per-generation (Keightley *et al.* 2009). A total of 10^5^ simulations was performed for each scenario, and summary statistics were calculated using functions from the *libsequence* library (Thornton 2003). Summary statistics and polymorphism measurements for all simulated evolutionary scenarios are reported in Table S3.

**Table 1:** Ten natural population surveys of *SR* chromosomes frequencies

Publication	*n*	Sex ratio chromosomes	BM only recombinants	T only recombinants	Standard arrangements
Sturtevant and Dobzhansky (1936)	416	0.135	0	0	0.865
Dobzhansky and Queal (1938)	1071	0.100	0	0	0.900
Koller (1939)	175	0.160	0	0	0.840
Dobzhansky (1939)	224	0.138	0	0	0.862
Dobzhansky (1943)	10495	0.149	0	0	0.851
Dobzhansky (1944)*	5753	0.096	0	0	0.904
Wallace (1948)	3866	0.124	0	0.001	0.875
Dobzhansky (1958)*	2214	0.140	0	0	0.860
Policansky (1974)	1561	0.195	0	0	0.805
Beckenbach (1996)	684	0.193	0.001	0.003	0.803
Total	26459	0.13455	0.00004	0.00015	0.86526

Tabulated are the total number of individuals genotyped (*n*) and the frequencies of *SR* chromosomes, recombinants carrying the basal and medial inversions only (BM), recombinants carrying the terminal inversion only (T), and Standard Arrangement. Linkage disequilibrium statistics from this table are *D* = 0.116 and *r^2^* = 0.998. Asteriks denote publications that include previously published samples, to avoid pseudo-replication a sample is only counted from its initial publication.

### Analysis of LD of the *D. pseudoobscura* SR chromosome

As a result of our pooled sequencing design, individual haplotypes could not be constructed from the assembled Illumina reads. Therefore, we designed PCR primers (Table S3) to amplify intergenic regions located on *XL*, and inside and outside of the inversions on *XR*. The chromosomal locations and approximate coordinates of the sequences are:

XL1 - XL; XL_group1a:2,958,187-2,959,179
XR1 - proximal of the basal inversion; XR_group6:370,850-371,767
XR2 - inside basal inversion; XR_group6:3,450,538-3,451,504
XR3 - distal of basal inversion/proximal of medial inversion; XR_group6:4,760,237-4,761,215
XR4 - inside medial inversion; XR_group6:9,392,822-9,393,842
XR5 - distal of medial inversion/proximal of distal inversion; XR_group8:2,908,477-2,909,427
XR6 - inside distal inversion; XR_group3a:327,359-328,353
XR7 - distal of distal inversion; XR_group5:349,989-350,987

We amplified the intergenic regions of eight *ST* strains and eight *SR* strains using PCR. We then directly Sanger sequenced the amplicons using the same primers. The sequences for each of the regions were aligned, and indels and singletons were removed for the analysis of LD. Segregating sites from each region were concatenated into a single sequence, and LD was estimated using the correlation based method of Zaykin *et al.* (2008). For each site, we also performed a Fisher’s exact test to determine the significance of allele association with *ST* or *SR*. Significance values were corrected for multiple testing using the Benjamini and Hochberg (1995) (BH) procedure.

### RNA collection

We isolated RNA from testes of six biological replicates of *SR* and *ST* fly strains. For each biological replicate, we pooled tissue dissected from testes from between 40 and 50 individuals. Individuals for each strain were maintained in three separate technical replicate growth chambers containing standard cornmeal-agar-molasses food media with yeast. The pooled testis tissue was immediately snap-frozen in liquid nitrogen after dissection and stored at −80° prior to RNA extraction. RNA was purified with RNeasy spin-columns (Qiagen) using the manufacturer’s instructions and stored at −80° before performing RNA sequencing. Total RNA concentrations for each sample were quantified using a nanodrop (Thermo Scientific).

### RNA-seq

Illumina RNA-Seq (Wang *et al.* 2009) was performed following standard protocols by the Baylor College of Medicine Human Genome Sequencing Center (Houston, TX) on an Illumina HiSeq 2000 sequencing platform. Briefly, poly-A+ mRNA was extracted from 1 μg total RNA using Oligo (dT)25 Dynabeads (Cat. No. 61002; Life Technologies) followed by fragmentation of the mRNA by heat at 94° for 3 min (for samples with RIN = 3–6) or 4 min (for samples with RIN of ≥6.0). First-strand cDNA was synthesized using the Superscript III reverse transcriptase (Cat. No. 18080-044; Life Technologies) and purified using Agencourt RNAClean XP beads (Cat. No. A63987; Beckman Coulter). During second-strand cDNA synthesis, dNTP mix containing dUTP was used to introduce strand-specificity. For Illumina paired-end library construction, the resultant cDNA was processed through end-repair and A-tailing, ligated with Illumina PE adapters, and then digested with 10 units of uracil-DNA glycosylase (Cat. No. M0280L); NEB). Amplification of the libraries was performed for 13 PCR cycles using the Phusion High-Fidelity PCR Master Mix (Cat. No. M0531L, NEB); 6-bp molecular barcodes were also incorporated during this PCR amplification. These libraries were then purified with Agencourt AMPure XP beads after each enzymatic reaction, and after quantification using the Agilent Bioanalyzer 2100 DNA Chip 7500 (Cat. No. 5067-1506), libraries were pooled in equimolar amounts for sequencing. Sequencing was performed on Illumina HiSeq2000s generating 100-bp paired-end reads. RNA-Seq Accession Numbers in the SRA database: (ST Biosample Numbers: SAMN06208344–SAMN06208349; SR Biosample Numbers: SAMN06208350–SAMN06208355).

### Read mapping and analysis of differential gene expression

The reads generated from RNA-Seq were mapped to the *D. pseudoobscura* reference genome (v. 3.2) (Thurmond *et al.* 2019) using the *subjunc* aligner (v. 1.4.6) under default parameters (Liao *et al.* 2013). As recommended in the user’s manual, read ends were not trimmed before aligning to the reference genome because the software soft clips ends with low mapping quality (MAPQ) scores. In total, over 755 million read pairs were generated. Between 33.2 million and 96.8 million reads were produced for each individual replicate (Table S5). An average of 81.9% of reads mapped to annotated features in the *D. pseudoobscura* reference genome. There was not a significant difference in the fractions of reads that mapped successfully between *SR* or *ST* replicates (82.0% and 81.7% respectively). Using *featureCounts* (v. 1.4.6), the number of reads mapping to each annotated exon were counted. We filtered out genes that did not have a minimum of 10 reads mapped in at least three individuals. After removing genes from the data that did not meet our filtering criteria, 14,687 genes were retained for analysis; 2247 of these genes are located on scaffolds mapping to *XR*.

After filtering, upper-quartile between-lane normalization was performed using the R package *EDASeq* (Risso *et al.* 2011). The read counts were further normalized using the *RUVs* method implemented in *RUVSeq* (Risso *et al.* 2014). *RUVs* is a normalization procedure to control for unwanted variation not associated with the biological covariates of interest (here, *SR* or *ST*) in the data. The factors of unwanted variation were estimated from the genes within each replicate group (*ST* and *SR*) because no differential expression is expected between chromosomes carrying the same arrangement. Normalization factors were estimated using the “relative log expression” (RLE) method of Anders and Huber (2010).

Differential gene expression was investigated in the normalized read counts using the R package *edgeR* (v. 3.10.2; Robinson *et al.* 2010). The covariates of interest (*i.e.*, *X*-chromosome arrangement) and the first factor of unwanted variation (k = 1) were used to construct the design matrix of the negative binomial generalized linear model (GLM). Briefly, the GLM takes the form of

logE[Y−W,X,O]=Wα+Xβ+O

where *Y* is the matrix containing the read counts for each gene, *W* is the matrix containing the factors of “unwanted variation”, *X* is the matrix containing the covariates of interest, and *O* is a matrix of offsets estimated through upper-quartile normalization. α and β indicate the parameters for the factors of unwanted variation and covariates of interest (*i.e.*, “treatment effect”, here the *X*-chromosome arrangement), respectively.

To test for significant differential expression between *ST* and *SR* males, a quasi-likelihood (QL) F-test was performed as implemented in *edgeR* with the *glmQLFTest()* function. The QL F-test is preferred to a standard likelihood ratio test because it reflects the uncertainty in dispersion estimates for each gene, and is a more robust and reliable method to control for the error rate (Lund *et al.* 2012). To correct for multiple testing, we corrected the raw *p*-values using the BH method (Benjamini and Hochberg 1995). We considered genes with a false discovery rate (FDR) < 0.05 as significantly differentially expressed (see Table S8 for a complete list of raw and corrected *p*-values for all genes)

### Analysis of recombination rates between medial and terminal inversions

To directly test for recombination in the collinear region between medial and terminal inversions of *D. pseudoobscura SR* chromosomes, we conducted a series of well-controlled testcrosses. Three independent *SR* chromosomes sampled from Zion National Park were isolated and background replaced by a minimum of seven generations of backcrossing to an isogenic stock. This isogenic stock carries the visible mutations *sepia*^*1*^ (se*^1^*, 1–145.1 marking the basal and medial inversions) as well as *short^1^* [*sh^1^*, 1–225.9 marking the terminal inversion; Orr and Irving (2001)], and has undergone more than seven generations of full-sib mating in the Phadnis laboratory prior to use in experimental crosses.

The recombination experiments follow the standard mapping conditions of Bridges and Brehme (1944) modified for the life-history and reproductive biology of *D. pseudoobscura*. In this case, 20 virgin females heterozygous the markers were collected over a 7 day period, aged for an additional 7 days, crossed to 20 males of the tester strain (*se*^*1*^* sh^1^*) under light CO_2_ anesthesia, allowed 24 hr to recover, and then tap transferred into milk bottles with 50 ml of standard cornmeal-molasses medium. The egg-laying period lasted 7 days, after which adults were removed from bottles and 0.5% v/v propionic acid was used to hydrate food as necessary. Emerging progeny were scored for visible markers daily starting from day 20 until the last individuals eclosed, only male progeny were scored because variable expression of the wing vein mutation *sh^1^* was observed in females. The experiment was conducted at room temperature without controlling for relative humidity or light/dark cycle.

The recombination experiment was conducted as a single-block, fully randomized design, with experimenter blind to treatment. A total of 33 experimental bottles were setup, consisting of 10 replicate bottles for each of the *SR* chromosome isolates in the heterozygous state and 3 additional bottles with *ST/ST* heterozygotes to calibrate our estimated genetic distances under these experimental conditions. The recombination rates for *se* and *sh* in the standard arrangement are so high that, after correcting for interference and multiple crossover events with Kosambi’s (1943) function, the genetic map distance exceeds the maximum limit of detection in a two point testcross (> 50 cM). In contrast, the extremely low recombination rate from all 10 bottles of each *SR* chromosome isolate required the data were pooled and reported with an exact binomial 95% CI. Recombinants, as determined by visible markers, were subsequently confirmed by scoring the presence/absence of the medial and terminal inversions of *SR* chromosomes via polytene chromosomes squash. A chi-square test for reciprocal classes of recombinants was conducted using the 1:1 Mendelian expectation.

The population genetic consequences of the estimated recombination rate is analyzed with a decay of gametic phase disequilibrium model (Crow and Kimura 1970, p. 47–50). To establish LD in natural populations, data from 10 published surveys, some containing samples from multiple localities and multiple times points, was compiled. These studies span 60 years and multiple investigators; however, the frequency of *SR* chromosomes was always estimated using polytene chromosome squashes of progeny from wild-caught inseminated females or wild-caught males crossed to a standard strain. The coefficient of LD, $D_{\left(AB\right)}$ is calculated as the deviation from observed haplotype frequency $P_{\left(AB\right)}$ from the expected haplotype frequency under random union of gametes with no physical linkage $\left(p_{A}q_{B}\right)$ (Gillespie 2004 p.140).

### Male germline segregation assay of recombinant SR chromosomes

To study the genetic architecture of *SR* chromosome drive, a male germline segregation assay was conducted. Genetic material for this assay consisted of reciprocal recombinants generated from one of the three *SR* chromosome isolates from Zion National Park used in the recombination experiment (*SR* Chromosome Isolate 2). This *SR* isolate was background replaced by a minimum of seven generations of backcrossing to an isogenic stock. This stock carries the visible mutations *cut*^*1*^ (ct*^1^*, 1–22.5), *sepia*^*1*^ (se*^1^*, 1–145.1 marking the basal and medial inversions), *lanceolet^1^* (*ll^1^*, 1–182.6, *snapt^1^* (sp*^1^*, 1–200.3), and *tilted^1^* (tt*^1^*, 1–228.2), the latter two mutations marking the terminal inversion (Orr and Irving 2001). This stock was obtained from the Species Stock Center (#14011-0121.08) and has undergone more than seven generations of full-sib mating in the Phadnis laboratory prior to use in experimental crosses. Recombinants of this *SR* chromosome isolate were obtained by scoring visible markers, backcrossing to the multiply marked chromosome, and polytene chromosome squash to confirm that crossing-over between inversions and not gene conversion at the mutant loci was responsible for the linkage phase change in visible markers.

The segregation assay follows similar conditions as the recombination experiments adjusted to vials instead of bottles. In this case, five virgin females homozygous for the markers were collected over a 7 day period, aged for an additional 7 days, crossed to five males of recombinant genotypes, standard arrangement (negative control), or nonrecombinant *SR* chromosomes (positive control) under light CO_2_ anesthesia. These crossed adults were allowed 24 hr to recover, and then tap transferred into fresh vials with standard cornmeal-molasses medium. The egg laying period lasted 7 days, after which adults were removed from bottles and 0.5% v/v propionic acid was used to hydrate food as necessary. Emerging progeny were scored for sex daily starting from day 20 until the last individuals eclosed, The experiment was conducted at room temperature without controlling for relative humidity or light/dark cycle. This segregation distortion phenotype is presented as the proportion of female progeny (*k*), with exact 95% binomial CIs.

### Data availability statement

The authors state that all data necessary for confirming the conclusions presented in the article are represented fully within the article. Custom Python code used to estimate FST and dXY as well as all R scripts used for plotting and statistical analyses are available at https://github.com/zfuller5280/Dpse_SR_analyses. Genomic sequences are deposited on the NCBI Short Read Archive (SRA) with accession numbers SRR6331544 and SRR6331545. RNA-Seq Accession Numbers in the SRA database: (*ST* Biosample Numbers: SAMN06208344-SAMN06208349; *SR* Biosample Numbers: SAMN06208350-SAMN06208355). The sequences of the breakpoints for the basal and medial inversions are contained in the Supplementary Text. Supplemental material available at figshare: https://doi.org/10.25386/genetics.12728543.

## Results

### Identification of *SR* chromosomal inversion breakpoints

**Figure 1:**
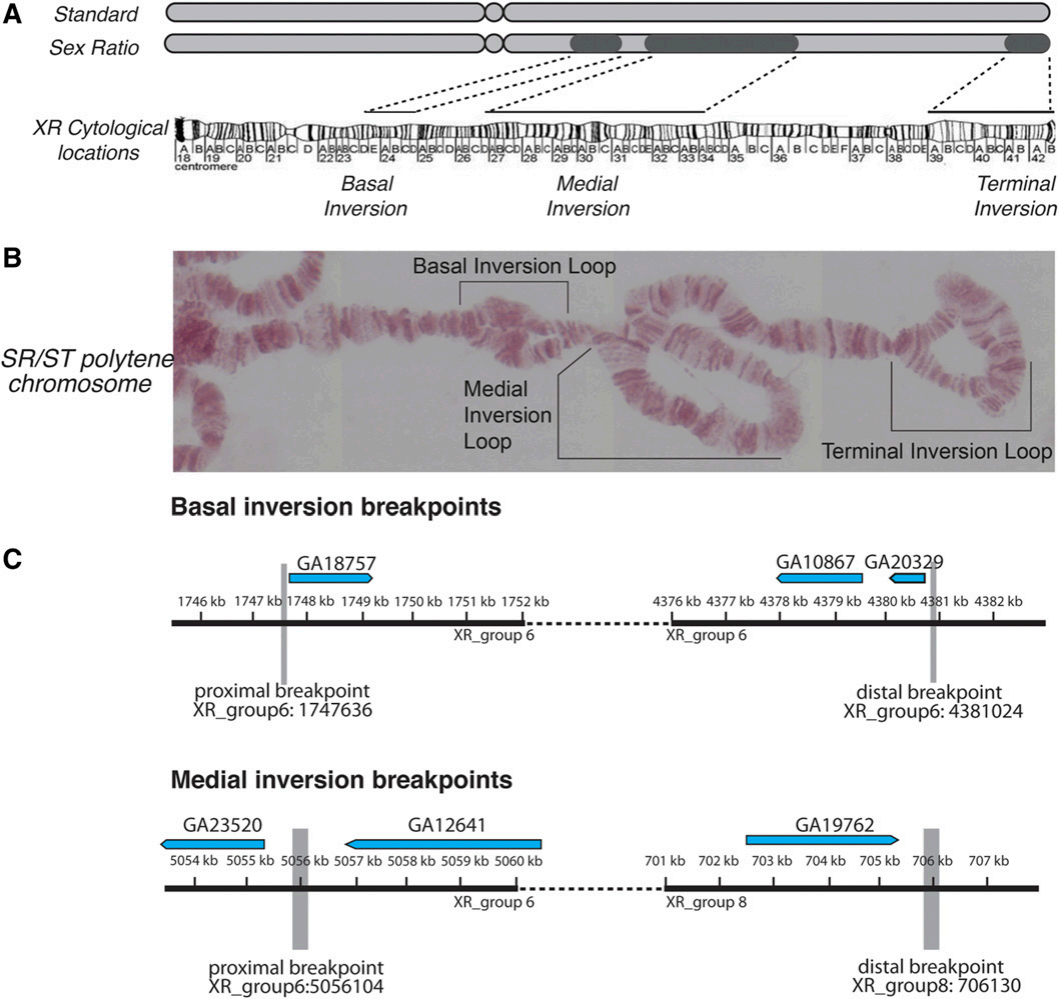
The structure of the *Sex-Ratio* (*SR*) chromosome and inversion breakpoints. (A) A schematic of the standard (*ST*) and *SR X*-chromosomes, with the darker regions showing the approximate locations of the basal, medial, and terminal inversions. The dotted lines show the locations of the three nonoverlapping inversions on the cytological map (Schaeffer *et al.* 2008) and polytene image of a *SR*/*ST* heterozygote female. (B) The genomic location and size of the basal inversion breakpoints. The coding regions of adjacent genes are shown above. (C) The genomic location and size of the medial inversion breakpoints. Similarly, the coding regions of adjacent genes are shown above.

To investigate the population genetics of the *SR* chromosome in *D. pseudoobscura*, we collected wild flies from Zion National Park (UT) and screened them for males that display strong sex ratio distortion. We isolated eight stably distorting stocks that produce >95% female progeny, and confirmed the presence of the three *SR* associated inversions with polytene chromosome analyses (Figure 1). Previously, the breakpoints of these inversions were coarsely mapped to major sections on the polytene maps (basal: sections 22 and 24; medial sections 24 and 33; terminal: sections 39 to 42) (Dobzhansky 1944; Beckenbach 1996), but are now mapped to subsections (basal: section 23D to 24D; medial: section 25D to 34A; and terminal: section 39A to 42B) (Schaeffer *et al.* 2008). We pooled DNA from eight independent *SR* lines and eight *ST* lines, performed whole genome resequencing with Illumina, and aligned the paired-end reads to the *D. pseudoobscura* reference genome (v.3.2) (Thurmond *et al.* 2019). While pooled sequencing precludes analyses that require haplotype information or individual sequence alignments, it offers a cost-effective approach to investigate patterns of polymorphism and allele frequencies, as well as to detect structural variants that differ between pools (Cutler and Jensen 2010; Kofler *et al.* 2011a,b; Hivert *et al.* 2018). Guided by the coarse locations on the chromosomal maps, we searched for read-pairs from the *SR* strains that aligned in the same orientation, yet in different regions of the chromosome were separated by large distances (>1 Mb) (Schaeffer *et al.* 2008; Corbett-Detig *et al.* 2012; Fuller *et al.* 2017). By scanning through these aberrantly mapped reads, we were able to identify candidate positions for two of the three pairs of inversion breakpoints.

Using PCR amplification followed by Sanger sequencing, we confirmed both the proximal and distal breakpoints of the basal and medial inversions (see Supplemental Material for locations, sequences, and additional description). However, we were unable to precisely locate breakpoints of the terminal inversion due to its proximity to the telomere, which consists of large blocks of repetitive sequences. Therefore, we use approximate cytological locations for the terminal inversion breakpoints in all subsequent analyses.

### Estimating the relative ages of the *SR* chromosome inversions

We used 250 kb regions centered at the inversion breakpoints to estimate the divergence between the *SR* and *ST* arrangements (see Tables S1 and S2 for details on the next generation sequence alignment statistics). Patterns of genetic divergence in regions immediately adjacent to inversion breakpoints are expected to preserve more information about the evolutionary origin and age of *SR* chromosome rearrangements than central regions of the inversion where gene flux resulting from double cross-overs or gene conversion is more likely (Navarro *et al.* 1997, 2000; Andolfatto *et al.* 1999; Matzkin *et al.* 2005; Noor *et al.* 2007; Wallace *et al.* 2011; Fuller *et al.* 2017, 2018). Across these regions, we estimated *F_ST_* at polymorphic sites (for intraspecies comparisons) and absolute sequence divergence (*d_xy_*) to *D. miranda* with CIs estimated by bootstrapping (Nei and Li 1979; Nei 1987; Hivert *et al.* 2018). Between the *SR* and *ST* arrangements we observed high overall levels of differentiation with a mean *F_ST_* of 0.761 (95% CI: 0.749–0.774). Using the transformation of Cavalli-Sforza (1969) and scaling to a speciation time of 2 MYA between *D. pseudoobscura* and *D. miranda*, we estimate this corresponds to a divergence time of 813 KYA (95% CI: 785–842) for the *SR* and *ST* arrangements. This falls within the range of the divergence time estimate obtained by Babcock and Anderson (1996) and precedes the 500 KYA estimated divergence time of *D. pseudoobscura* and its sister species *D. persimilis* (Babcock and Anderson 1996; Wang and Hey 1996; Hey and Nielsen 2004; Noor *et al.* 2007; Fuller *et al.* 2018). Between *ST* and *D. miranda*, *d_XY_* was estimated as 1.26 × 10^−2^ (95% CI: 1.25–0.1.27 × 10^−2^) across the breakpoints. A higher level of absolute sequence divergence is observed for *SR* and *D. miranda* in these same regions, with *d_XY_* estimated as 1.41 × 10^−2^ (95% CI: 1.40–1.42 × 10^−2^).

We next compared estimates of differentiation for each set of inversion breakpoints individually to infer the order of their formation on the *SR* chromosome. *F_ST_* is similar for regions surrounding the basal (*F_ST_*: 0.803, 95% CI: 0.793–0.814) and medial (*F_ST_*: 0.785, 95% CI: 0.773–0.796) inversion breakpoints, as the 95% CIs overlap. In regions flanking the terminal inversion breakpoints, *F_ST_* is lower (0.696; 95% CI: 0.682–0.711) compared to the medial and basal breakpoints, providing evidence that the terminal inversion is the youngest on the *SR* chromosome. However, we also note the caveat that the terminal inversion breakpoints are the most coarsely mapped.

Although the terminal inversion breakpoints are less differentiated between types than either the basal or medial inversions, the overall high levels of differentiation suggest it is still quite old. Using the same transformation of *F_ST_*, we estimate the age of the terminal inversion to be ∼708 KYA (95% CI: 682–738), which is toward the lower end of the range of the divergence time estimated by Babcock and Anderson (1996) and still predates the divergence of *D. pseudoobscura* and *D. persimilis* (Noor *et al.* 2007). This result is consistent with all three inversions being present in the ancestral species or soon after the split with *D. persimilis*, providing further evidence for the role of ancestral polymorphisms in explaining observed patterns of divergence and differentiation (Fuller *et al.* 2018).

**Figure 2:**
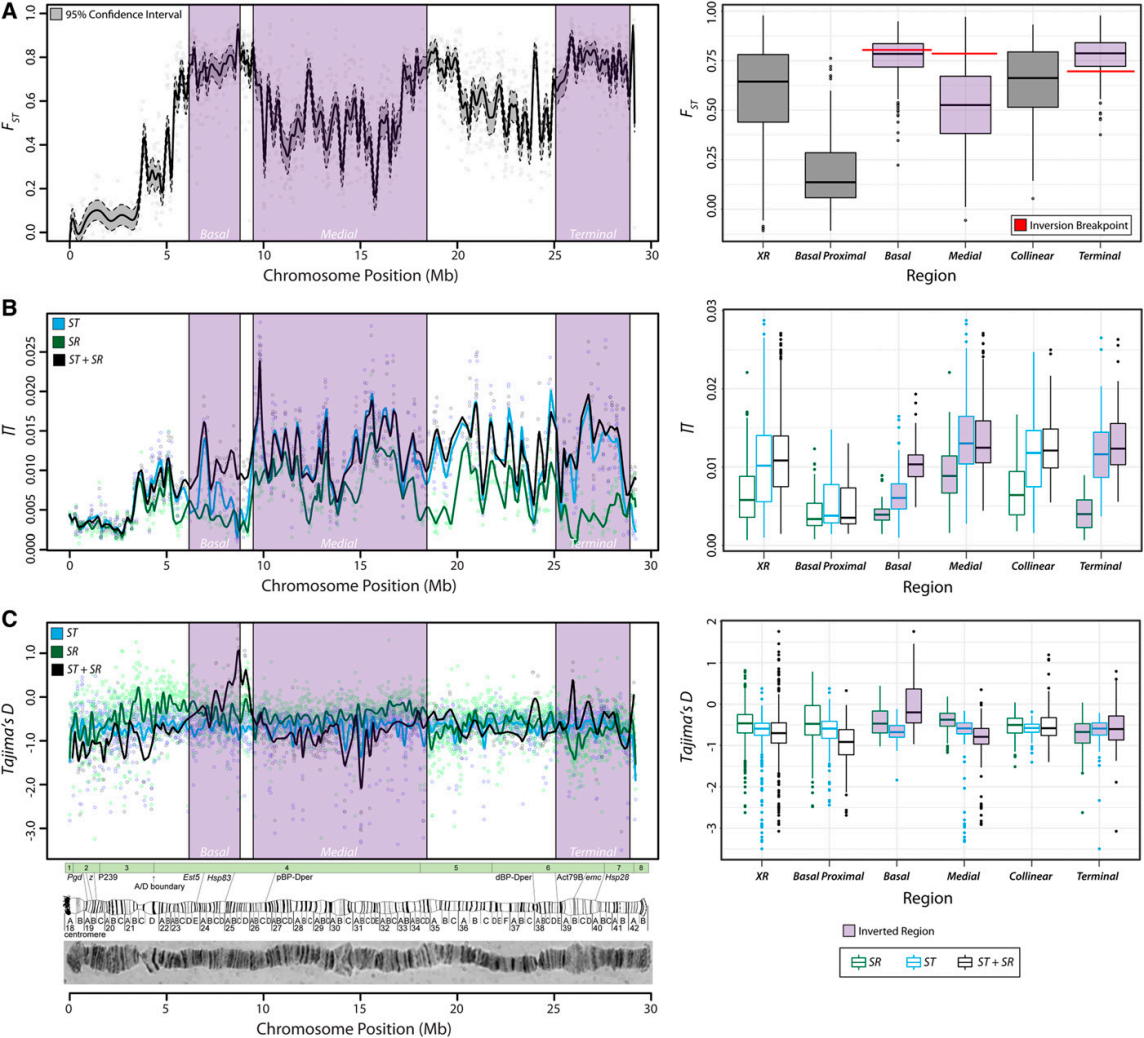
Elevated genetic differentiation and patterns of polymorphism across *XR*. (A) *F_ST_* was estimated in nonoverlapping sliding windows of 100 SNPs across chromosome *XR*. Gray dots show the estimate of *F_ST_* in each window. The black line represents the loess smoothed average *F_ST_* across the chromosome and the gray region bounded by dotted lines is the loess smoothed average 95% bootstrapped confidence interval of the mean estimate of *F_ST_* within each window. Purple shaded regions indicate the locations of the basal, medial, and terminal inversions. The boxplots on the right shows the distribution of *F_ST_* summarized for different regions of the chromosome, indicated on the *x*-axis. (B) Nucleotide diversity measured as the mean proportion of pairwise differences (*π*) in 10-kb windows of intergenic regions for *ST* (blue), *SR* (green), and both arrangements jointly (black). Each colored line shows the loess smoothed average across the chromosome, and dots represent each window. The boxplot on the right shows the distribution of *π* summarized for different regions of the chromosome, indicated on the *x*-axis. (C) The site frequency spectrum summarized with Tajima’s *D* in the same nonoverlapping 10-kb intergenic windows. Lines and dots are colored consistently with the plot above. The boxplots on the right shows the distribution of *D* summarized for different regions of the chromosome, indicated on the *x*-axis. Below, polytene images of each chromosome and sketches of the cytogenetic regions with the approximate locations of common genetic markers are depicted below each plot. The green boxes represent the linear ordering and size of genomic scaffolds used to construct the chromosome sequence from Schaeffer *et al.* (2008).

If we extend our analysis of differentiation by estimating *F_ST_* in nonoverlapping sliding windows of 100 SNPs across the entire length of *XR* it can be demonstrated that high levels of genetic differentiation are not restricted to the breakpoints and instead remain elevated across each inversion (Figure 2A). Even in the 6.6 Mb collinear region between the medial and terminal inversion, where single or double crossovers should form and reduce levels of genetic differentiation relative to inverted segments, *F_ST_* is not significantly less than within inverted regions (Mann-Whitney *U*-test, *p* < 0.99; Figure 2A). Together, these results demonstrate a high level of genetic differentiation between *SR* and *ST* that extends across the three overlapping inversions, and indicate that the terminal inversion is likely the youngest on the chromosome.

### Patterns of polymorphism, fixed differences, and long range LD on the *SR* chromosome

The observed pattern of genetic differentiation suggests that recombination has been effectively suppressed between arrangements for at least the last 700 KY in regions spanning the three inversions. While restricted genetic exchange has led to the accumulation of differences between *SR* and *ST*, these differences can arise from a number of nonmutually exclusive mechanisms. First, neutral differences may accumulate as the result of new mutations arising within an arrangement and then increasing in frequency by drift, particularly within *SR* as it is presumed to have a smaller long-term effective population size (*N_e_*) based on current observed frequencies. Second, positive selection acting on modifiers of *SR* could generate recurrent hitchhiking events over the course of long-term genetic conflict, thereby reducing overall levels of polymorphism, yet rapidly increasing the frequencies of linked sites unique to *SR*. Finally, non-neutral population dynamics or other selective forces, such as epistatic selection or associative overdominance, may allow for mutations to accumulate in an old polymorphism such as *SR* that do not follow simple neutral expectations. We next examined patterns of polymorphism within each type to investigate and test these evolutionary scenarios.

We measured nucleotide diversity as the average proportion of pairwise differences (*π*) within *ST* and *SR*, as well as jointly across both types (Figure 2B). To examine patterns of polymorphism at putatively neutral sites, we restricted the analysis to only intergenic regions and estimated *π* in 10 kb nonoverlapping windows. Across the chromosome, *SR* has reduced levels of diversity compared to *ST*, although they are most similar in the region proximal to the basal inversion (*π_ST_* = 5.25 × 10^−3^, *π_SR_* = 4.20 × 10^−3^; Mann-Whitney *U*-test, *p* = 0.016). Chromosome wide, there is nearly a 1.6x reduction in diversity observed on *SR*, with a mean *π* of 6.5 × 10^−3^ (95% CI: 6.3–6.7 × 10^−3^) compared to a mean *π* of 1.03 × 10^−2^ (95% CI: 1.00–1.06 × 10^−2^) on *ST*. The greatest reduction in diversity is found across the terminal inversion, where *π* is ∼2.8x lower in *SR* compared to *ST* (*π_ST_* = 1.18 × 10^−2^, *π_SR_* = 4.14 × 10^−3^; Mann-Whitney *U*-test, *p* < 2 × 10^−16^). Although diversity is reduced in *SR* compared to *ST*, this reduction in diversity is not as low as expected under a simple neutral coalescent model. To generate neutral expectations of diversity in *SR* relative to *ST*, we simulated coalescent histories and considered a range of scenarios for the long-term *N_e_* of *SR*, spanning a minimum of 1% to a maximum of 30% of the frequency in the total population. In simulations where the *N_e_* of *SR* was 1% of the population, this reduction was >100x (see Table S3). Regardless of the scenario considered, the mean reduction in diversity of *SR* relative to *ST* was consistently lower than 4x and the neutral coalescent model was rejected at a significance level of 5% (Table S3). Thus, the observed level of polymorphism across the *SR* chromosome rejects a simple neutral model of divergence, mutation, and drift as the sole evolutionary forces shaping patterns of genetic diversity.

We next examined distortions in the site frequency spectrum that may result from recurrent hitchhiking or large-scale sweeps by selection acting on drivers, enhancers, or suppressors-of-suppressors. In the same 10 kb intergenic windows, we summarized the site frequency spectrum with Tajima’s *D* (Tajima 1989) for both *SR* and *ST* (Figure 2C). The site frequency spectrum of *SR* does not show a significant skew toward rare variants that would be expected in the case of large scale hard selective sweeps or hitchhiking events removing polymorphism across vast genomic regions (*D_SR_* = −0.499; 95% CI: −0.523 to −0.478). In fact, the chromosome wide average of *D* is more positive on *SR* compared to *ST* (*D_ST_* = −0.669; 95% CI: −0.694 to −0.648), indicating there is less of a skew toward rare variants on *SR*. Moreover, there are no large scale distortions in the frequency spectrum observed for any of the three *SR* associated inversions or intervening collinear regions, as the average *D* > −1 within each (Figure 2C). The qualitative conclusions do not change if all regions of the chromosome are included in the analysis (*i.e.*, windows with protein-coding genes are used as well; see Figure S3, A and B). We also considered the possibility that such selection may reduce polymorphism only within protein coding genes and lead to differences in diversity observed at nonsynonymous sites. Thus, we estimated *π* per-synonymous and per-nonsynonymous site (*i.e.*, *π_S_* and *π_N_*) for all nonoverlapping complete genes in *ST* and *SR* to examine differences in polymorphism within coding sequences. No significant differences in *π_N_* were detected between *ST* and *SR* in any inverted or collinear region of the chromosome. Furthermore, the only significant differences in *π_S_* were observed in the genes located within the terminal inversion and the intervening collinear regions, although the average reduction was less than twofold in each (Figure S4). Together, these results reject a scenario where recurrent hitchhiking or selective sweeps are the primary forces shaping genetic diversity on *SR*.

**Figure 3:**
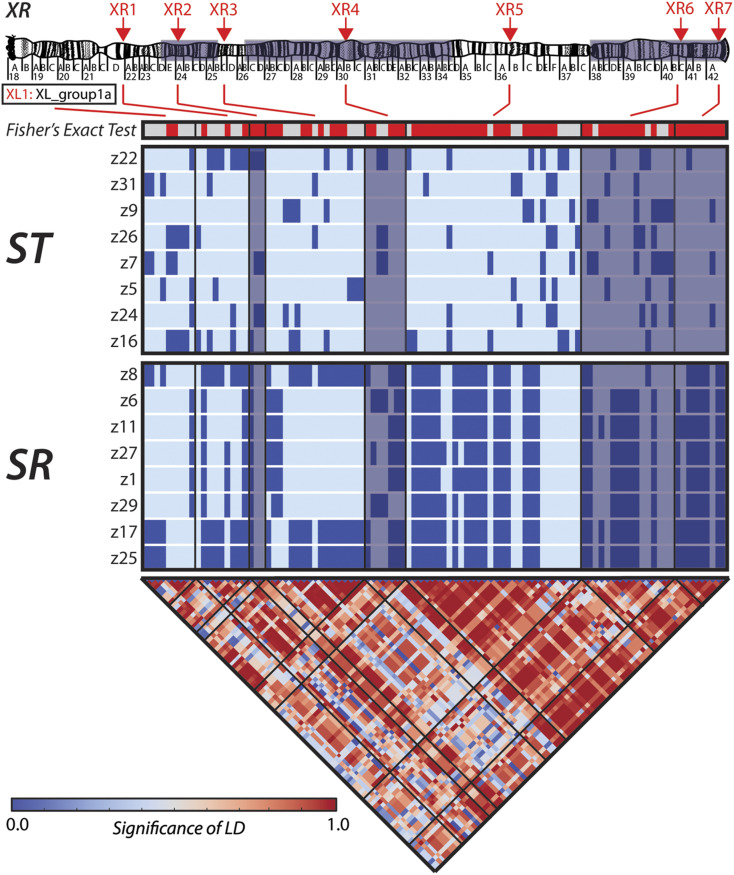
Long-range linkage disequilibrium (LD) is present across *XR*. LD was estimated using the PCR-amplified sequences of eight intergenic markers. The red arrows at the top of the chromosome sketch (Schaeffer *et al.* 2008) show the approximate location of each sequenced marker. The single horizontal bar depicts the results (*P* < 0.05 = red, *P* ≥ 0.05 gray) of a Fisher’s exact test for the association between alleles and chromosome (*SR* and *ST*) type. The following horizontal bars show haplotype diagrams for polymorphic sites in the sequenced *ST* (top) and *SR* (bottom) strains, with darker colored sites representing the derived allele. The bottom triangular heat map shows the significance of LD for all polymorphic sites in the sequenced intergenic regions estimated with the correlation-based approach of Zaykin *et al.* (2008). Red indicates greater LD and blue represents nonsignificant allele associations. The black lines show the boundaries between each intergenic region.

Higher than expected levels of diversity and slightly more positive Tajima’s *D* on *SR* relative to *ST* has also been observed on the *SR X*-chromosome of *D. neotestacea* (Pieper and Dyer 2016). Because no long range LD or excess of fixed differences were observed in *D. neotestacea*, this pattern of polymorphism on *SR* was explained by occasional gene flow with *ST*, which is also thought to prevent the accumulation of deleterious mutations (Pieper and Dyer 2016). Therefore, to explore this possibility in *D. pseudoobscura*, we next investigated patterns of LD across the chromosome and compared levels of fixed differences to shared polymorphisms. To examine LD between segregating sites across the chromosome, we designed PCR primers to amplify eight intergenic regions on the *X*-chromosome (Table S4). We individually sequenced these regions from all eight strains of both *SR* and *ST*, and concatenated the sequences to perform the LD analysis (Figure 3). The power of Fisher’s Exact Test (FET) to detect significant LD depends on the sample size and allele frequencies at the two loci (Brown 1975). Of the pairwise comparisons of SNPs capable of rejecting the hypothesis of linkage equilibrium with FET (Lewontin 1995), 10% of them show significant LD. Moreover, in contrast to what has been observed in *D. neotestacea*, there are significant associations detected that span several Mb across the chromosome, indicating the presence of long-range LD. Likewise in contrast to *D. neotestacea*, there are multiple derived sites within the amplified intergenic regions that are fixed within *SR* and not observed in *ST*.

By analyzing fixed derived SNPs in our pooled sample of *SR* chromosomes, we can examine the pattern of alleles that are in absolute LD (*i.e.*, where LD is at its maximum value) with one another in our sampled strains. After determining the ancestral state with *D. miranda*, there are a total of 43,579 sites that are fixed for a derived allele unique to *SR* chromosomes. We next estimated the proportion of such fixed derived sites in 10 kb windows, finding a chromosome wide mean of 2.21 × 10^−3^ (95% CI: 2.119–2.3 × 10^−3^). In contrast, a total of only 71 such sites are found on the entirety of *XL*, although we note this number may partially represent an artifact of our crossing scheme (see Supplemental text). For comparison, the chromosome wide mean proportion for derived alleles fixed in *ST*, yet not observed in *SR* is 1.47 × 10^−4^ (95% CI: 0.893–2.05 × 10^−4^). The fixed differences in *SR* are found predominantly across the regions of the chromosome where recombination is likely the most suppressed. In the collinear region proximal to the basal inversion, where recombination can presumably still occur, the mean ratio of fixed differences in *SR* to shared polymorphisms is 0.015. However, the ratio of fixed differences to shared polymorphisms is substantially higher across the three inversions, with means of 1.72, 0.468, and 0.801, for the basal, medial, and terminal, respectively. Moreover, this ratio is similarly high for the intervening collinear regions, with a mean value of 0.813. While the number of fixed differences is high across the regions of the chromosome experiencing suppressed recombination, nonetheless we observe shared polymorphisms in all regions. These shared polymorphisms, however, are not driving the pattern of the site frequency spectrum in *SR*, evidenced by the average Tajima’s *D* remaining > –1 across all regions when they are removed from the analysis (Figure S3C). Thus, while we cannot exclude the possibility of occasional exchange with *ST*, gene flow between arrangements does not appear to be the predominant force determining patterns of polymorphism on *SR*, as a large number of fixed differences have accumulated and long range LD is observed.

Together, these results reject a simple neutral model of mutation and divergence as well as a model of recurrent hitchhiking in explaining the maintenance of genetic differences between *SR* and *ST*. Moreover, the presence of excess fixed differences and long-range LD across the inverted regions indicate that occasional gene flow via recombination between arrangements is not the primary force shaping patterns of polymorphism. Instead, these results support a more complex scenario in which other selective forces, such as epistatic selection, act to maintain genetic differences.

### The *SR* arrangement provides a massive mutational target

A subset of the fixed differences held together in almost perfect association likely lead to functional changes contributing directly to the *SR* phenotype. It is also possible that some of these functional changes may be responsible for mitigating the effect of autosomal suppressors and *Y*-linked resistance, allowing the *SR* trait to persist for >2 million generations.

To identify potential targets for both the evolution of distorters and their enhancers, we first determined which genes contain fixed amino acid differences. Of the total number of derived sites fixed in *SR*, 7221 occur within protein coding regions, including 2612 nonsynonymous changes found across 905 genes. This corresponds to over 30% of all genes on *XR* containing at least one fixed amino acid difference between *SR* and *ST*. A total of 17 fixed sites specific to *SR* are predicted to be loss-of-function mutations because they introduce premature stop codons or disrupt splice sites. Together, these results indicate that a substantial number of potentially protein altering changes are harbored on the *SR* chromosome, some of which may have deleterious effects on fitness. While the *SR* phenotype may be highly polygenic, many of these amino acid changes could be unrelated to the distortion phenotype, and instead exist in tight linkage with the *SR* trait as a consequence of suppressed recombination and high differentiation of the chromosome.

**Figure 4:**
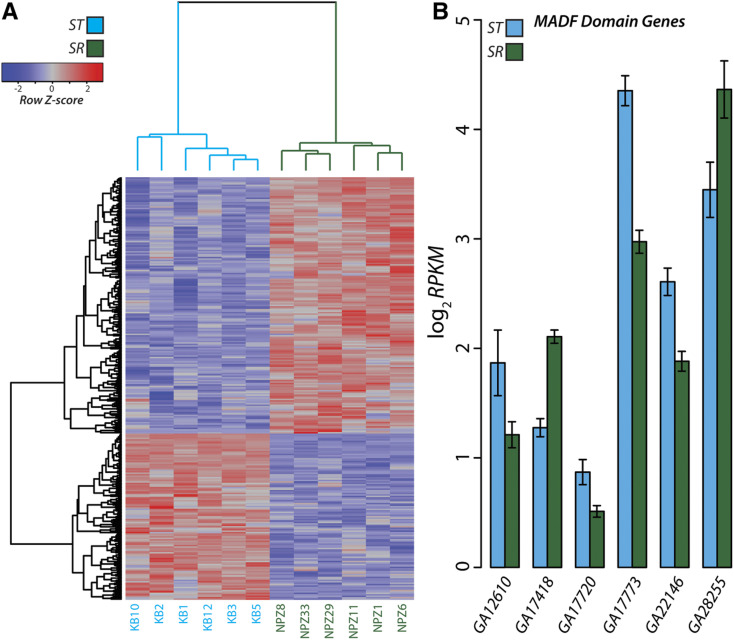
Differentially expressed genes across *XR*. (A) Differential expression depicted as a heat map for the 292 significant (*q* < 0.05) genes on *XR*. The individual strains (columns) and genes (rows) are arranged according to unsupervised hierarchical clustering. Each gene is colored according to the deviation from the mean level of expression across all individuals. (B) Expression levels for the 6 MADF domain containing genes detected as significantly differentially expressed. The height of each bar represents the mean expression for *ST* and *SR* measured as reads per kilobase per million mapped reads (RPKM) with error bars indicating the standard error.

In addition to amino acid changes, functional effects may result from changes in patterns of gene expression. We therefore performed RNA-seq on tissue dissected from testes to test for significant expression level differences between *ST* and *SR* (see Tables S4 and S5 for RNA-Seq read data and differential expression statistics). Because of the crossing scheme used to maintain the strains, we restrict our analysis to genes located on the *X*-chromosome (see Supplemental Material for a further discussion). In total, we detected 292 significantly differentially expressed on the right arm of the chromosome (Figure 4A). For differentially expressed genes on *XR*, there is an enrichment of those upregulated (177) relative to *ST* than those that show lower expression (115; Fisher’s Exact Test: *p* < 0.009).

We performed a gene ontology (GO) analysis using DAVID software to test for the enrichment of common biological functions, pathways, and protein domains among genes we detected as differentially expressed (Huang *et al.* 2009). After correcting for multiple testing, remarkably the only category that remains significantly enriched among the 335 differentially expressed genes on the *X*-chromosome contain MADF domains (*q* < 0.041, BH corrected). A number of MADF domain containing genes have previously been associated with segregation distortion in *D. pseudoobscura* and other *Drosophila* species (Hutter and Ashburner 1987; Orr and Irving 2001; Barbash and Ashburner 2003; Barbash *et al.* 2003; Phadnis and Orr 2009). Here, this category contains six genes (Figure 4B) that are all located on *XR*. Four of the genes (*GA17720*, *GA17773*, *GA22146*, *GA28255*) harbor multiple fixed amino acid changes unique to *SR* with the *D. melanogaster* orthologs to *CG11723* and *stwl* containing 15 and 17, respectively. Additionally, *GA17720* and *GA22146* both contain multiple fixed intronic differences. In combination with the previously described role of MADF domains in segregation distortion, this set of differentially expressed genes are attractive candidates for follow-up studies to dissect the molecular basis of the *SR* trait.

It is possible that some of these differentially expressed genes result from artifactual *trans*-acting autosomal differences generated from our crossing design. Thus, to further narrow down a set of candidate genes among those differentially expressed on *XR*, we retained transcripts containing at least one fixed nucleotide difference between *SR* and *ST*. In total, 224 genes of the 292 differentially expressed on *XR* also contain a minimum of one fixed difference. Of these, 123 have at least one fixed nonsynonymous change (Table S7). Moreover, 50 genes contain at least one fixed difference in a 5′-untranslated region, splice site or intron, providing possible *cis*-regulatory changes responsible for the observed expression differences (Table S7). However, it is also possible the underlying changes for other genes exist in unannotated promoter or *cis*-regulatory regions. While no significant GO enrichments were detected among these more restricted sets of differentially expressed genes on *XR*, they represent promising candidates for future work focused on understanding the functional genetic basis of the *SR* phenotype.

### SR inversions strongly suppress recombination across the entire chromosome arm

We hypothesized that this extensive genetic differentiation across the chromosome could be due to either completely suppressed recombination in these regions or epistatic selection acting on linked inversions (Sturtevant and Beadle 1936; Roberts 1976). Directly addressing the former hypothesis, a cytogenetic analysis of 107 and 96 offspring from two female heterozygotes (*ST*/*SR*) found no evidence for recombination between the medial and terminal inversions (see Supplemental Information for methods and results). These data suggested that, if crossing over happens between the medial and terminal inversion, it occurs at a frequency <1% (Tables S8 and S9).

**Figure 5:**
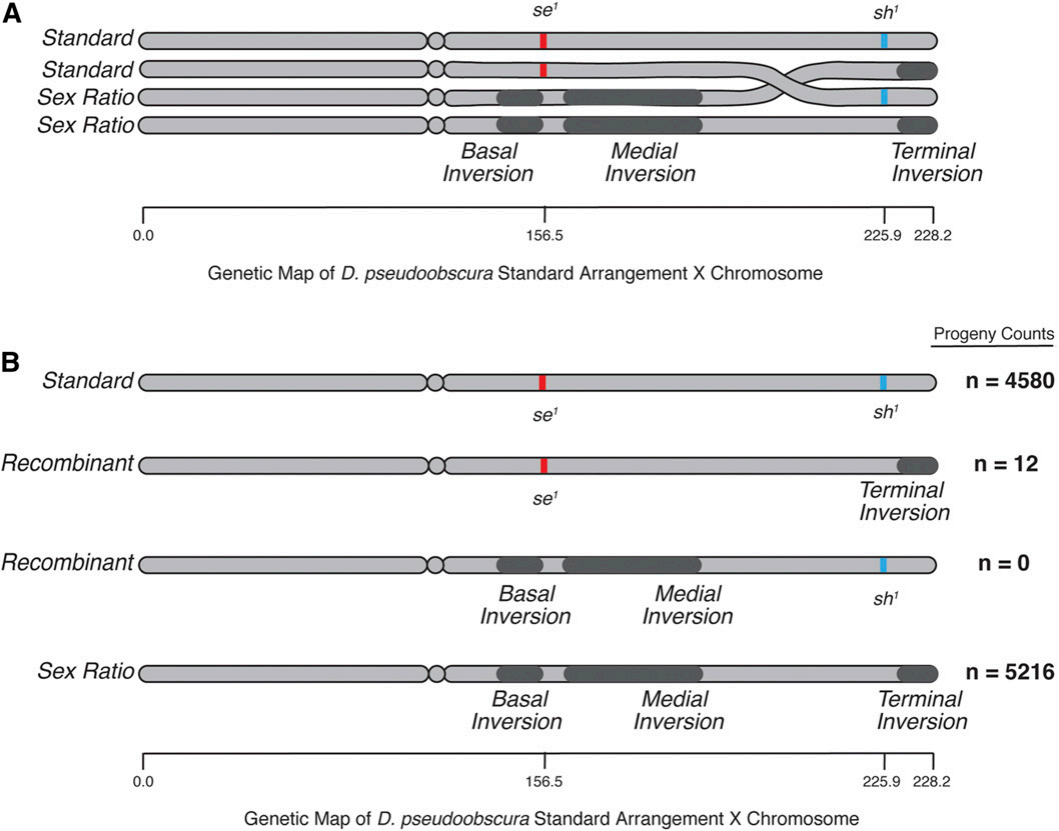
Overview of recombination experiments of LD. (A) Illustration of the four-strand bundle present in prophase of Meiosis I for a *SR/ST* heterozygote. Depicted in red and blue are the relative positions of visible mutations to inversions of the *SR* chromosome (∼70 cM apart on the standard arrangement genetic map). The position of both markers and inversions relative to the standard arrangement genetic map is approximate and not exact because inversion heterozygosity strongly distort this map. (B) The four possible chromosomes recovered in the recombination experiment, with the pooled progeny counts recorded to the right, please note the complete absence of one reciprocal class of recombinants (basal and medial inversions with visible marker *sh^1^*).

The collinear region of 6.6 Mb between the medial and terminal inversions is between 40 and 50 cM on the standard genetic map (Chovnick 1973; Orr and Irving 2001; McGaugh *et al.* 2012), and models developed for *D. melanogaster* of genetic flux with inversions incorporating crossover interference suggest the crossover rate in this region should be 0.01–0.001 events per meiosis (Navarro *et al.* 1997; Koury 2018). Consistent with these interference models, rare *SR* recombinants have sometimes been observed in nature (Wallace 1948; Beckenbach 1996). Therefore, we performed a second recombination experiment using three independently sampled *SR* chromosomes isolated on a multiply marked standard arrangement genetic background to detect rare recombination events on the order of 10^−4^ per meiosis. Gene conversion at marker loci could be misinterpreted as rare recombination events; however, gene conversion events occur on the order of 10^−5^ per site per meiosis, and are, therefore, an order of magnitude lower than the designed level of detection in our experiment (Chovnick 1973; Korunes and Noor 2019). The isogenic stock used for background replacement carried mutants of *sepia* (*se*^*1*^, 156.5 m.u. marking the basal and medial inversions) and *short* (*sh^1^*, 225.9 m.u. marking the terminal inversion); therefore recombination, or lack thereof, can be directly assayed with standard testcrossing procedures (Figure 5A).

In this second recombination experiment, a total of 10,891 progeny were scored from 33 experimental bottles, 10 replicate bottles for each of three *SR* isolates and 3 replicates of a single *ST* gene arrangement. The recombination fraction observed between *se* and *sh* in *ST* arrangement homozygotes was 0.4224; with Kosambi (1943) correction this translates to a genetic distance >50 cM, consistent with previous observation (Orr and Irving 2001). From all *SR/ST* heterozygotes, only 12 recombinant chromosomes were recovered, yielding an estimated genetic distance of 0.12 cM and an approximate 500-fold decrease in recombination in the collinear region of *SR* chromosomes (Figure 5B, Table S9). This recombination fraction is consistent with the lower values predicted by the *D. melanogaster*-based interference model of recombination suppression for inversion heterozygotes (Navarro *et al.* 1997; Koury 2018). Interestingly, 12 of 12 recombinants carried only the terminal inversion, while none of the reciprocal class (basal and medial inversion only) were recovered. This is a very unexpected result ($\chi_{\left[1\right]}^{2}=12$, *p* < 2.85 × 10^−4^) (Table S10), suggesting that, in addition to strong recombination suppression, there is also selection acting on linked inversions.

Although the observed recombination rate is low, it is sufficient to cause complete dissociation between the inversions on either side of the large collinear region on a short evolutionary timescale. To quantitatively understand the interaction of low levels of recombination (on the order of 10^−3^ per meiosis) over long periods of evolution (on the order of 10^6^ generations), we consider a simple model consisting of two loci that evolve in a random mating population of infinite size without epistasis. After *t* generations, the LD between them will be $D_{t}={\left(1-c\right)}^{t}D_{0},$ where *D_0_* is the initial disequilibrium and *c* is the recombination rate from our experiment (0.0012) (Gillespie 2004, p. 140). Using six decades of *SR* chromosome frequency data from surveys of natural populations of *D. pseudoobscura* to establish an initial *SR* frequency of 0.135 with initial disequilibrium of 0.116 (Table 1), LD half-life in this model is only 577 generations, with effective linkage equilibrium achieved within 10,000 generations, demonstrating that suppressed recombination alone cannot account for the near-perfect association observed after millions of generations of recombination without the action of epistatic selection. Haploid selection coefficients (*s*) were introduced to this model as the proportion reduction in fitness for each gametic type (*ST* Arrangement chromosome, Full *SR* chromosome, Basal and Medial Inversion Recombinant *SR* chromosome, and Terminal Inversion only Recombinant *SR* chromosome) assuming the wildtype *ST* arrangement chromosome has fitness of 1. This approach requires extreme selection to reconcile laboratory recombination rates and the relative rarity of *SR* recombinant chromosomes in nature: *s* = 0.32 for the terminal inversion-only recombinant and *s* = 0.65 for the basal and medial inversions recombinant (see Supplementary Methods and Results for full derivation of the model and fit of haploid selection coefficients).

### Ongoing selection maintains the epistatic genetic architecture underlying strong *SR* chromosome drive

After establishing the feasibility of generating *SR* chromosome recombinants in the laboratory, further efforts (>10,000 males screened) produced both of the reciprocal recombinants from a single *SR* chromosome isolate on an isogenic genetic background (Species Stock Center 14011-0121.08 containing mutants *ct*^*1*^ *se*^*1*^* ll^1^ sp^1^ tt^1^* for screening recombinants). Previous analysis of the rare recombinants sampled from nature only qualitatively classified the recombinants as nondriving (terminal inversion only), or as drivers (basal and medial inversions only), before the stocks were lost or discarded (Wallace 1948; Beckenbach 1996). With some difficulty, laboratory stocks of our recombinants can be established and maintained as segregating lines (see Figure S7 for polytene squashes).

**Figure 6:**
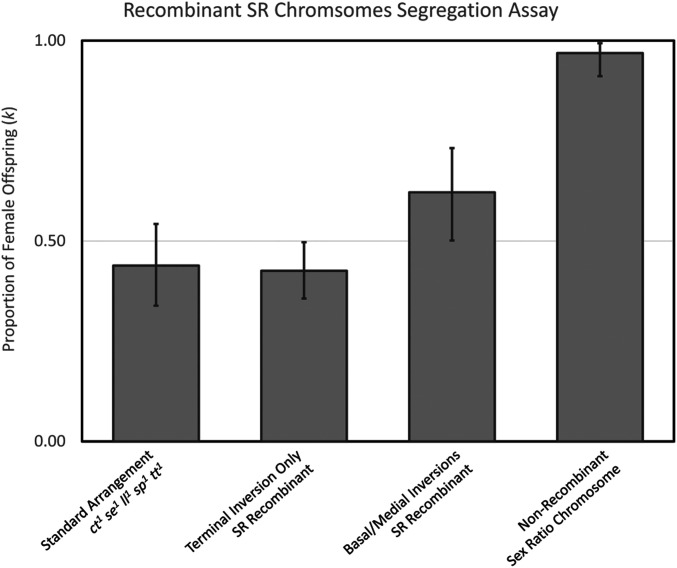
Segregation assay of recombinant *SR* chromosomes. Strength of the distortion phenotype is expressed as a proportion of female offspring for both reciprocal recombinants. Non-recombinant standard arrangement and full *SR* chromosomes are included as negative and positive controls, respectively. Error bars represent exact 95% confidence intervals based on binomial distribution.

To investigate the genetic variation underlying *SR* chromosome drive, we performed a male germline segregation assay for each of the reciprocal recombinants using both the nonrecombinant *SR* chromosome and the standard arrangement as positive and negative controls, respectively (Figure 6). Consistent with previous reports (Wallace 1948; Beckenbach 1996), the terminal inversion only *SR* recombinant did not exhibit drive (*k* = 0.426, exact 95% binomial confidence interval: 0.357–0.497) when compared to the standard arrangement (*k* = 0.439, exact 95% confidence interval: 0.3339–0.547). In contrast, the basal and medial inversion carrying *SR* chromosome recombinant is capable of driving (*k* = 0.622, exact 95% confidence interval: 0.501–0.732), but drive is strongly reduced compared to the nonrecombinant *SR* chromosome (*k* = 0.969, exact 95% confidence interval: 0.911–0.994). These results suggest an epistatic genetic architecture of at least two loci; minimally, a causal drive locus residing in the proximal half of the right arm of the *X*-chromosome with a modifier locus in the distal half of the chromosome arm that either directly strengthens the driving locus or acts as a suppressor of a suppressor.

To incorporate the drive phenotypes of *SR* chromosomes and their rare recombinants into the theoretical treatment of the decay of LD requires modeling sex-specific genotypes in a diploid population. In this case, the classical model for the decay of gametic phase disequilibrium presented above is not appropriate for *SR* chromosomes for at least two reasons First, *X*-linked segregation distorters cause a sustained difference in allele frequencies between the sexes, invalidating the random union of gametes assumption (Charlesworth and Hartl 1978). Second, the *SR* chromosome system is presumed to be maintained in natural populations under drive-selection balance with major fitness defects associated with *SR* chromosomes (Curtsinger and Feldman 1980). To address these concerns, we produce a more realistic model of gametic and genotypic frequencies by incorporating *X*-linkage, male-specific segregation distortion, female-specific recombination, sex differences in allele frequency, and enforcing equilibrium drive-selection conditions (see Supplementary Methods and Results for derivation and iteration of the formal equations for this model).

Under drive-selection conditions, and assuming the same initial disequilibrium and recombination rate, linkage between the *SR* chromosome terminal inversion and the basal/medial inversions decays even faster than the neutral gametic model (LD half-life is 47 generations, with equilibrium achieved within 200 generations). Accordingly, intense selection against recombinant *SR* chromosomes is necessary to prevent their rapid accumulation in natural populations. To incorporate this, we added standard viability selection to the model, assuming all fitness defects are recessive and independent for the four homozygous genotypes (see Supplemental Methods and Results). In addition to the 43% homozygous fitness reduction required to balance strong male germline drive of the full three-inversion *SR* chromosome, numerical analysis suggests at least a 1% homozygous fitness decrease is necessary for the *SR* recombinant carrying the terminal inversion, whereas the basal and medial inversion *SR* recombinant must have at least a 29% homozygous fitness decrease to prevent wholly replacing the full three-inversion state *SR* chromosome. While we do not believe either the simple neutral results of the previous section or the more complex numerical analysis reported here fully capture all the biologically relevant fitness components, we note that under different genetic models (haploid *vs.* diploid) and different assumptions (random union of gametes *vs.* drive-selection balance) the results qualitatively agree. These results suggest that in the absence of selection against recombinants of the *SR* chromosome, the near perfect association of *SR* chromosome inversions in natural populations should have broken up long ago, *F_ST_* in (as well as LD across) the collinear regions should be substantially lower, and recombinant *SR* chromosomes should be found at appreciable frequencies (*q* > 0.12) in present-day populations. Together, our direct experiments and population genetic models show that both suppressed recombination and strong selection against recombinants are required to maintain the three-inversion *SR* chromosome state in natural populations.

## Discussion

The *SR* system of *D. pseudoobscura* has historically served as an important example of segregation distortion and still presents many unanswered questions for the evolution of *SR* chromosomes (Sturtevant and Dobzhansky 1936; Wallace 1948; Beckenbach 1996; Price *et al.* 2019). Here, through population genomic analyses and direct experiments, we demonstrate that a combination of extensive recombination suppression and strong epistatic selection act to maintain the highly differentiated *D. pseudoobscura SR* chromosome.

First, we used polymorphisms surrounding inversion breakpoints on *SR* chromosomes to estimate their ages. In agreement with previous phylogenetic analyses of the *Esterase-5* gene indicating ancient monophyletic origins of the *SR* chromosome in the ancestral species of *D. pseudoobscura* and *D. persimilis* (Babcock and Anderson 1996), we estimate the basal and medial inversions to have arisen ∼800,000 years ago. The same analysis revealed the terminal inversion is younger, arising ∼100,000 years later, and, importantly, this younger regions contains a modifier locus necessary for the full manifestation of strong *SR* distortion (Figure 6). This first result shows segregation distorters can be so old that they may even predate the divergence of the species that carry them, and can continue to accrue new inversions and modifiers of distortion as they evolve. The long-term persistence and continuing maintenance of the *D. pseudoobscura SR* chromosome adds to a growing number of observed old selfish meiotic drive elements maintained in current populations across diverse taxa (Lyttle 1991; Lindholm *et al.* 2016; Kelemen and Vicoso 2017). For example, in mice, the *t*-haplotype distortion system has been maintained over long evolutionary timescales, even in the face of occasional recombination, which has the potential to reduce drive through the homogenization of alleles between wild and *t*-haplotypes (Kelemen and Vicoso 2017).

Our estimate for the age of the inversions was inferred from the high levels of differentiation in recombination restricted regions surrounding the inversion breakpoints. Second, we extended our investigation to the pattern of polymorphism, differentiation, and LD that extended well beyond the inversion breakpoint boundaries and across intervening collinear regions spanning >80% of *XR*. At this scale, *SR* chromosomes harbor more diversity than expected when compared against simulated, neutral coalescent histories; therefore, we reject mutation and drift as the sole forces generating these patterns. The site frequency spectrum revealed that *SR* chromosomes have a slight excess of rare variants (*i.e.*, negative Tajima’s *D*) but are less skewed toward rare variants than *ST* chromosomes (Figure 2C). Therefore, we reject the recurrent sweeps model for the evolution of *SR* chromosomes. We find an excess of fixed differences on and strong long-range disequilibrium across *SR* chromosomes (Figure 3), supporting extensive recombination suppression and further ruling out occasional gene flow via recombination as the primary force shaping patterns of polymorphism. Although we cannot specify the dynamic selective and demographic history of *D. pseudoobscura SR* chromosomes, we note that similar patterns in polymorphism have been observed in *SR* chromosomes of *D. neotestacea* (Pieper and Dyer 2016), we suggest these commonalities may stem from being the minority allele in an ancient chromosome-wide balanced polymorphism. Further comparative population genetic studies are needed to understand the different evolutionary forces acting on these *SR* chromosomes that yield similar patterns of polymorphism.

The three *SR* chromosome inversions have generated a large highly differentiated region spanning >80% of *XR*, containing >2100 genes, and to tease out which genetic differences are responsible for the *SR* phenotype will be challenging. Our third result identified >500 genes harboring multiple fixed derived amino acid differences and >200 genes have significantly differentially expressed transcripts on the *SR* chromosome. There are, however, some loci that are intriguing candidates to explore further. For instance, the gene (GA28653) with the largest number of fixed amino acid changes (21) is the ortholog to *Spc105R* in *D. melanogaster*, which produces a kinetochore protein that is required for the co-orientation of sister centromeres during meiosis and promotes the accurate segregation of chromosomes (Radford *et al.* 2015). Moreover, MADF domain containing genes are significantly enriched for differential expression, which are also implicated in hybrid-incompatibilities in *D. melanogaster* and its closely related species *D. mauritiana*, *D. simulans*, and *D. sechellia* (Hutter and Ashburner 1987; Barbash and Ashburner 2003; Barbash *et al.* 2003; Maheshwari *et al.* 2008). Notably, another gene product containing a MADF domain is *Overdrive* (*Ovd*), which has previously been identified as a single gene that underlies both male sterility and segregation distortion in hybrids between the USA and Bogota subspecies of *D. pseudoobscura* (Orr and Irving 2005; Phadnis and Orr 2009). Furthermore, our results may also contain candidate loci that have become associated with the primary distorting alleles because they act as enhancers, or impede the action, of suppressors.

Inversions are well known suppressors of recombination (Sturtevant 1921; Roberts 1976), and the gametic loss of products from single crossovers initiated within inverted regions is well established (Sturtevant and Beadle 1936; Novitski and Braver 1954). Over many generations, and across evolutionary timescales, due to the lack of the homogenizing force of recombination, increased genetic differentiation between alternative karyotypes is often a consequence of inversions. Thus, our finding of genetic differentiation within and outside of inverted segments is consistent with the general reduction of genetic exchange expected in the presence of the three nonoverlapping inversions in *SR* chromosomes. Population genetic analyses have revealed similar effects of suppressed recombination on patterns of genetic diversity within and outside inverted segments in several other *Drosophila* species (Machado *et al.* 2007; Noor *et al.* 2007; Stevison *et al.* 2011; McGaugh and Noor 2012; Corbett-Detig and Hartl 2012; Fuller *et al.* 2017) In *Drosophila* systems with nonoverlapping inversions, suppressed recombination has also been observed to extend across large intervening collinear regions of the chromosome (Singh and Singh 1988; Kumar and Gupta 1991; Mestres *et al.* 1998; Munté *et al.* 2005). Based on the pattern of elevated chromosome-wide divergence and paucity of recombinant chromosomes in the wild, it is tempting to attribute the lack of gene flow between *ST* and *SR* chromosomes to the suppressive effects of the inversions alone. However, in our fourth result we critically demonstrated through direct experimentation that recombination between *ST* and *SR* chromosomes does occur; and in absence of selection against *SR* recombinants, crossing-over occurs at a rate more than sufficient to rapidly dissociate the terminal inversion from the basal and medial inversions in natural populations.

We estimate that recombination occurs at a rate on the order of 10^−3^ per meiosis between the medial and terminal inversions. While low, using both a simple two locus model and a more realistic numerical simulation, we demonstrate this recombination rate is sufficient to have achieved complete linkage equilibrium well within the lifetime of the *SR* chromosome. By comparing the observed frequencies of recombinant chromosomes in nature to their expected frequencies, we estimate appreciable haploid selection coefficients acting against recombinants (*s* = 0.316 and *s* = 0.649, for terminal inversion only recombinants and basal/medial inversions recombinant, respectively). Modeling haploid selection coefficients does not discriminate how this selection is manifested and represents the cumulative effects of viability, fertility, virility, and gametic selection integrated over all life-history stages and in both sexes. Interestingly, prior experimental analysis has shown reduced viability and fertility of *SR* homozygous females (Wallace 1948; Curtsinger and Feldman 1980; Larner *et al.* 2019), suggesting a large portion of the selection is realized in females and not directly related to the segregation distortion mechanism. In other segregation distortion systems, direct male fitness costs have been identified, for example, fewer offspring are produced by *SR* males carriers in populations of *D. simulans* and males heterozygous for *SD* in *D. melanogaster* have reduced fertility, although in *D. pseudoobscura* hemizygous *SR* male virility is normal, despite failure of half the sperm to develop (Hartl *et al.* 1967; Policansky and Ellison 1970; Beckenbach 1981; Montchamp-Moreau and Cazemajor 2002) (see Table S9). In *D. pseudoobscura*, it remains an open question and active area of research to identify the fitness costs balancing the transmission advantage of *SR* chromosomes, with some authors arguing for reduced reproductive fitness in carrier males (Wedell 2013; Price *et al.* 2014), increased polyandry in females, differences in fertility between *SR* and *ST* males, and temperature-dependent virility reduction in *SR* (Policansky 1979; Wu 1983a,b; Price *et al.* 2010; Wedell 2013). Neither the simple model of LD decay with haploid selection coefficients nor the more complex numerical analysis explores all the modes of selection and fitness arrays that could produce such a uniquely long-lived chromosome-wide balanced polymorphism. This would require extensive fitness measurements, such as those undertaken by Wallace (1948), Curtsinger and Feldman (1980), and Larner *et al.* (2019). Instead, we model either all-inclusive haploid selection coefficients or simple viability selection on recessive factors, which qualitatively agree on the intense strength of ongoing selection to maintain the near perfect association of the three inversions in natural populations.

In our final result, we demonstrate the strong unsuppressed distortion observed in *D. pseudoobscura* is a product of epistatic interactions of at least two genes bound together by the inversions of the *SR* chromosome. Segregation assays of rare *SR* chromosome recombinants that separate the terminal inversion from the basal and medial inversions revealed a necessary and sufficient, but weak, distorting locus in the proximal half of the chromosome (Figure 6). The strong unsuppressed distortion phenotype is expressed only when this weak distorting locus is paired with a modifier locus in the distal half of the chromosome (Figure 6). Interestingly, our age estimates of the terminal inversion suggest the region that contains this modifier locus is younger (100,000 years) than the region containing the weakly distorting locus. Combining our first result on inversion ages with our last result on segregation of recombinant *SR* chromosomes, suggests a historical scenario where *D. pseudoobscura SR* chromosomes evolve stronger drive by accumulating modifiers and additional inversions to bind these epistatic alleles in tight coupling phase LD. Taken together with the old age and extensive differentiation present across the *D. pseudoobscura SR* chromosome, despite constant low rates of recombination, this indicates that strong epistatic selection has been persistent and pervasive over the lifetime of the *SR* chromosome.

Despite being one of the longest studied selfish chromosomes, many fundamental genetic and evolutionary aspects of the *D. pseudoobscura SR* chromosome have remained mysterious (Sturtevant and Dobzhansky 1936; Price *et al.* 2019). While theory predicts that distorting systems should be short-lived (Hamilton 1967; Vaz and Carvalho 2004), our results provide further evidence that the *D. pseudoobscura SR* system is surprisingly old (Babcock and Anderson 1996). Given the lack of detected suppressors and its relatively stable population frequencies over the last century of sampling, together these observations indicate other evolutionary forces must be acting on the *D. pseudoobscura SR* chromosome. Here, through population genomic analyses and direct experiments, we have demonstrated that the combination of strongly suppressed recombination and strong ongoing epistatic selection act to maintain the *SR* chromosome and its associated inversions as an extensively differentiated genomic region.
